# Using communities of practice in adult social care to build research capacity and foster best practice: a qualitative evaluation

**DOI:** 10.3310/nihropenres.14134.1

**Published:** 2025-12-19

**Authors:** Vanessa Abrahamson, Ferhana Hashem, Sophie Fournel, Collette Thornton, Wenjing Zhang, Olivia Trapp, Rasa Mikelyte, Liz Jones, Ann-Marie Towers

**Affiliations:** 1 Centre for Health Services Studies, University of Kent, Canterbury, England, UK; 2 Disability Assist, Nimbus Enterprise Park Liphook Way, Maidstone, UK; 3 Cartref Homes (UK) Ltd, Sittingbourne, Kent, ME10 1NA, UK; 4 HSCWRU, King's College London Faculty of Social Science & Public Policy, London, England, UK; 5 Innovation & Partnership, Adult Social Care & Health, Kent County Council, Maidstone, Kent, ME14 1XX, UK; 6 National Care Forum, Coventry, England, UK

**Keywords:** Social care, social work, research capacity, communities of practice, qualitative research, practitioner, care provider, local authority.

## Abstract

**Background:**

Communities of Practice (COP) are recognised as shared learning spaces that situate learning, deepen knowledge, and facilitate the exchange of expertise within a specific domain. While CoPs often emerge organically, they have been widely adopted across health, social care, and education. However, their civic potential, particularly in enabling people with lived experience of social care to collaborate with practitioners to shape practice and drive meaningful change, remains underexplored.

The Kent Research Partnership, South-East England (2021-5), aimed to build research capacity in adult social care. As part of its workstreams two COPs were co-designed with informal carers and people who draw on care and support. The themes (‘Complex needs’ and ‘Workforce’) were co-developed by a prioritisation exercise. Each COP had monthly online sessions with invited speakers and facilitated discussions. Participants included informal carers, people who draw on care/support, social care practitioners, researchers, and other people interested in the sector.

**Methods:**

This study evaluated the COPs and their contribution to research capacity building in Kent. Using a pragmatic approach, 21 participants were purposively selected and interviewed.

**Results:**

Reflexive thematic analysis generated three key themes: fostering an inclusive and collaborative learning environment; enabling shared learning within and beyond the COPs; and generating shared impact through influence on policy and practice. These findings were mapped against Cooke’s (2005) framework for building research capacity.

**Conclusions:**

Participants valued the inclusive and safe learning space, which supported mutual reflection and knowledge exchange. Relationships formed across roles and settings which helped bridge siloed thinking, validate research ideas, and extend learning beyond the COPs. Time constraints and organisational culture affected frontline social worker participation, despite a recognised need for innovation. Networking emerged as a prominent outcome, leading to new knowledge-exchange collaborations. Future research should focus on understanding the impact of the COPs on organisational level practice and policy.

## Plain Language Summary

This article explores how Communities of Practice (COPs), groups where people come together to learn from each other, can help improve adult social care. While COPs are often used in health and education, less attention has been paid to their role in supporting people with lived experience of social care to work alongside professionals and influence change.

The Kent Research Partnership (2021–2025) was a project set up to strengthen research in adult social care. Two Communities of Practice were created: one focused on supporting people with complex needs and the other on the adult social care workforce. Monthly online meetings included guest speakers and open discussions focused on research ideas and projects. Members included informal carers, people who draw on care and support, practitioners, researchers and other people interested in social care.

The study looked at how these groups helped build research skills and connections in adult social care. Interviews with 21 participants showed that people valued the welcoming and inclusive environment which encouraged open sharing and learning. The groups helped build relationships across different roles and organisations, allowing people to learn from each other and work together more effectively. Some challenges were noted, such as time pressures and workplace culture, which made it harder for some frontline staff to take part. However, many participants said the groups helped spark new ideas, build confidence and led to useful collaborations.

Overall, the Communities of Practice created a space where people with different experiences could come together, learn from each other, build relationships, break down barriers between organisations, and spark new ideas which could help shape better social care services. Future research should focus on understanding the impact of the COPs on practice and policy.

## Introduction

Until recently, Communities of Practice (COP) have largely been absent as a forum for learning for people with lived experience including informal carers (family, friends, husbands/wives providing care and/or support) and those needing care or support. However, recent developments have seen a proliferation of COPs that has enabled and encouraged idea exchanges and shared learning between these groups. In a climate beset by finite resources, COPs offer a learning space that moves beyond professionally managed learning to humanistic and democratic approaches, putting the group’s shared learning at the heart of knowledge acquisition, supporting epistemic justice ( Gray *et al.*, 2010) This paper begins by outlining the context for the re-emergence of COPs, first focusing on Wenger’s original discourse ( 1998), describing how COPs offer coherence in a practice community, underpinned by *mutual engagement*, *negotiation of a joint enterprise* and a *shared repertoire*. In recent years, COPs have been presented primarily as a hub for professional shared-learning in organisations. However, by returning to these concepts, we explore the constituent parts of a COP, to understand the internal processes and features of what makes COPs work ( Iverson & McPhee, 2008). We identify the benefits and impact of two COPs that were co-developed in adult social care, based in South-East England: one focused on supporting people with complex needs and the other on enhancing, diversifying and sustaining the social care workforce.

## Background

### Communities of Practice theory

Communities of Practice have been employed in social care for several years but more recently shifted from a professional to a community space to generate and share knowledge about social care practice and research ( Hashem *et al.*, 2024). The notion of COPs has been in existence for almost 30 years and was originally conceived as a mechanism to promote tacit forms of learning. Wenger (1998) argued that COPs have historically emerged organically, evolving into naturally occurring learning spaces. This typically occurs under conditions where tacit knowledge develops subconsciously within individuals and is further shaped as it becomes internalised and expressed as ‘common sense’. Wenger suggests that COPs are the prime context in which knowledge and knowledge-production are held ( Wenger, 1998; Wenger *et al.*, 2002).

**Figure 1.**
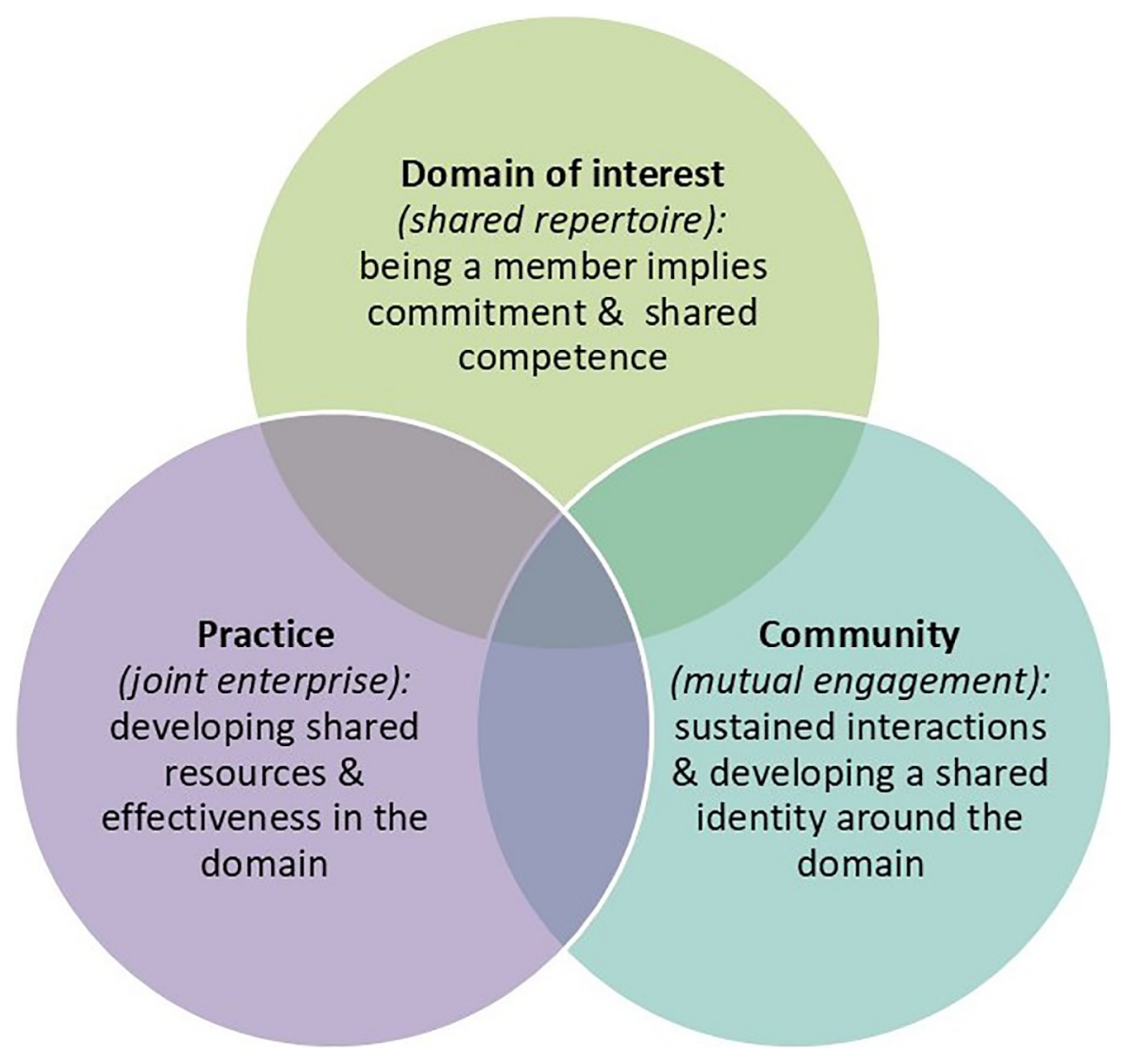
The three domains of a Community of Practice.

The constituents of COPs were re-configured from Wenger’s original discourse ( 1998) and re-worked around three fundamental elements that are now considered building blocks to every COP: a domain of knowledge, which defines a set of issues; a community of people who are concerned about this domain; and a shared practice whereby members are striving to be effective in their domain ( Wenger, 1998; Wenger *et al.*, 2002). Figure 1 demonstrates these constituents and the previous terminology. These three characteristics provide the ‘glue’ or supporting structures, connections and processes that keep COPs alive and active ( Iverson & Mcphee, 2002; Iverson & McPhee, 2008).

Over time, Wenger and colleagues defined a COP as a set of people who “share a concern, a set of problems, or a passion about a topic, who deepen their knowledge and expertise in this area by interacting on an ongoing basis” ( Wenger *et al.*, 2002). Wenger (1998) suggests that COPs have an inherent predisposition to deepen learning in a professional context, enabling learning to be embedded into the goals of an organisation. Wenger (1998) argued that COPs are an organisation’s assets, aimed at generating knowledge and being hard-wired into the fabric of an organisation, despite not being formal entities in themselves. The renaissance of COPs over the last 15 years, as a way for improving organisational performance, is apparent in areas including healthcare ( Ranmuthugala *et al.*, 2011), nursing ( Gullick & West, 2016), social work ( Gray *et al.*, 2010) and academic-practice partnerships ( Padilla & Kreider, 2020).

The conceptualisation of COPS aligns closely with Cooke’s (2005) framework for building research capacity in healthcare which outlines six interrelated principles: developing skills and confidence; ensuring research is close to practice; fostering linkages, partnerships and collaborations; supporting dissemination; promoting sustainability; and investing in infrastructure. COPs naturally support Cooke’s (2005) emphasis on collaboration and practice-based learning, offering a dynamic environment where research capacity can be nurtured through peer support, shared goals, and an egalitarian learning environment.

### Background to the COPs

The COPs evaluated in this paper were initially established to help build a research culture, inform ongoing research, and foster networks and collaborations that bring together research ideas across adult social care. This was an integral part of the Kent Research Partnership project (2021–5) based in South-East England, a collaboration with the local authority (a two-tier county council) which aimed to foster adult social care partnerships and build research capacity. It was funded by National Institute for Health and care Research (NIHR)’s Health and Social Care Delivery Research programme (HSDR; NIHR131373).

The process in which the COPs were formed is outlined elsewhere ( Hashem *et al.*, 2024) but included three engagement exercises with stakeholders, including informal carers and people using care and support (abbreviated to ‘care experienced’), to identify the focus (July and November 2021, and March 2022). Three key concepts underpinned the COPs: co-production; equality, diversity, inclusion and intersectionality; and practice-oriented approach. The formation and operationalisation of the COPs were planned in-person but due to the COVID-19 pandemic, the engagement exercises took place exclusively online and set the direction for the COPs to be an entirely online forum over the next three years. However, all COP members were invited to join a mid-point (September 2023) and final event (May 2025) for the overall Kent Research Partnership which were held in-person; following the mid-point event, new participants joined the COPs. Two COPs were established: (a) Supporting people with complex needs throughout the lifespan (shortened to ‘Complex Needs’), and (b) Enhancing, diversifying and sustaining the social care workforce (shortened to ‘Workforce’). Both COPS co-developed aims and anticipated outcomes ( Table 1).

**Table 1.** Aims and outcomes for COPs (co-developed).

**Aims**	1. Build a culture of research around issues relevant to the COP domain and community. 2. Inform ongoing research relevant to the COP domain. 3. Networking to collaborate on bringing research ideas together.
**Suggested outcomes**	1. Identify specific research topics and questions within the COP domain. 2. Support individuals / build teams to develop potential projects. 3. Submit new research funding applications. 4. Build resources (using a cloud-based platform, Glasscubes) on ‘pockets’ of topics for everyone to consult, and share within and beyond the COP. 5. Support individual learning and development opportunities including applications for research funding provided by Kent Research Partnership and elsewhere e.g. Applied Research Collaboration Kent Surrey Sussex.

Fifty-one COPs, including three joint ones, took place between June 2022 and April 2025. They were initially chaired by academics but this role was transferred to practitioners and a member of our Lived Experience Working Group, as part of democratising and trying to engage practitioners. Both COPs had the same format of a speaker on a specific topic relevant to its domain, followed by discussion. Initially, COPs were 1.5 hours, with two speakers but later were reduced to one hour and one speaker, aimed at increasing the attendance of frontline staff. Topics for discussions were initially driven by all participants, stipulating what topics would be helpful but as the momentum reduced, reflected in lower attendance, it fell to the Researchers in Residence - researchers embedded within the local authority partner’s adult social care team ( Smith *et al.*, 2025) - to find speakers. Topics ranged widely, with both COPs providing a forum for local researchers to present ideas and receive feedback. Examples of presenters at the Workforce COPs included representatives from strategic organisations including Skills for Care, East Kent Health and Care Partnership, National Care Forum and Kent Integrated Care Alliance. The Complex Needs COP had a wider remit including different groups (e.g. homelessness, dementia, young people leaving care, older people, learning disabilities) and a range of projects (e.g. social prescribing, footcare in care homes, enhanced technology, exercising).

Membership numbers remained high throughout, with 115 people signed up to the Complex Needs COP and 122 in the Workforce COP (with some individuals participating in both). To facilitate communication among COP members in between meetings, we also used a cloud-based platform (‘Glasscubes’) that enabled COP members to share communication and resources in one place, allowing discussion, and making it easy for COP members to contact each other. It had fewer members, 64 for Complex Needs and 69 for Workforce COP. Monthly meeting attendance was smaller, between 7023 attendees per meeting for Complex Needs and 8–28 for Workforce meetings; joint COPs had larger attendance of 30–35.

## Aims

The study aimed to evaluate the Kent Research Partnerships COPs and their impact on research capacity building in adult social care in Kent. Objectives were to:

1. Describe both CoPs including aims, membership and format.
2. Explore members’ views on being part of the COP and the benefits/opportunities this afforded.
3. Identify tangible short- and longer-term outcomes that contribute to research capacity building in adult social care.
4. Identify facilitators and barriers to successful implementation, running and sustainability of COPs in adult social care.

## Methods

We adopted a pragmatic research methodology, selected for its close alignment with the values of social work and the ethos of our COPs. Pragmatism in social science is grounded in the belief that knowledge is generated through action and experience, and that ideas must be tested and refined through their practical application ( Morgan, 2014). It embraces methodological flexibility, allowing researchers to draw on both qualitative and quantitative approaches to address complex, real-world problems. This aligns with the dynamic nature of social care settings and services, where contextually relevant and actionable insights are essential. Pragmatism also acknowledges that reality is both socially constructed and evolving, shaped by individual and collective experiences ( Creswell & Clark, 2017; Morgan, 2014). These principles resonate strongly with social work’s emphasis on experiential learning, reflection, and action for social justice ( Kaushik & Walsh, 2019), making pragmatism an appropriate paradigm for this evaluation.

### Patient and Public Involvement (PPI)

Kent Research Partnership had active PPI involvement and engagement throughout all aspects of the project, stemming from the overall project’s inception in 2021. A vibrant Lived Experience Working Group ( Mikelyte *et al.*, 2025) met monthly and remained active throughout, providing ongoing guidance at every stage. For example, the research questions were informed by their involvement in running and attending the COPS, and they contributed to decisions about the study design. As the study progressed beyond the design stage, the group continued to inform key elements of the evaluation through monthly discussions. This included input on the choice of interviews, recruitment strategies, and approaches to data analysis. Members of the Lived Experience Working Group also played an important role in disseminating findings through their connections with allied social care organisations and linked communities, such as the local authority’s PPI body. The study’s public co-applicant co-led the Working Group and chaired many of the Complex Needs COPs. In addition, our care-experienced co-author (CT), a regular COP member, was integral to sustaining and evaluating the COPs.

### Study design

A qualitative study design was employed using in-depth interviews to explore the influences and impacts of the COPs. Interviews were selected as a pragmatic approach to capturing the views of busy practitioners/stakeholders and unpack the ‘black box’ of how COPs worked, for whom and in this setting. Online interviews were the preferred format for most participants ( Bryman, 2016).

### Study participants

We used a purposive sampling strategy and participants were chosen based on predefined criteria that aligned with the purpose of the study ( Bryman, 2016). We wanted a balance of settings (e.g. social care, university, third sector) and disciplines including members of the public (those already involved in Kent Research Partnership and/or regular attendees, and less frequent attendees); practitioners who attended regularly, and/or have presented, or who stopped attending; researchers who attended regularly and/or presented at the COP; those in strategic roles. We emailed individuals using our mailing list (as we had prior consent to do so) with an invitation to participate as well as the participant information sheet and consent form ( Abrahamson, 2025) to complete and return by email. Written informed consent was taken from each participant and a time/date for interview was arranged, with the option of online or face-to-face.

### Data collection

The topic guide ( Table 2) was adapted from a previous study on COPs as the context was similar, in that both involved partnerships across several organisations ( Abrahamson *et al.*, 2025). The topic guides were split into three versions for practitioners, researchers and public contributors ( Abrahamson, 2025) and refined for each participant group. Overall, 21 interviews were carried out (by Author 1) in January and February 2025, with 16 online and five in-person. Online interviews were transcribed using Microsoft Teams automated transcription and reviewed by Author 1; face-to-face interviews were recorded with a digitally encrypted Dictaphone and transcribed by a transcription service.

**Table 2.** Interview guide - main areas.

**1. Background:** participants role, experience and involvement with COPs.
**2. Understanding COPs:** exploring participants understanding of COPs, e.g. purpose.
**3. COP engagement:** which COPs they attended and why, their role e.g. if they presented or Chaired
**4. Expectations, aspirations & contributions:** exploring expectations/aspirations, contributions, benefits of attending.
**5. Learning:** what was learnt, specifically any research skills e.g. developing a research question.
**6. Facilitation and leadership:** issues around how COPs are facilitated, inclusion, suggestions for improvement.
**7. Impact:** achievements, (unexpected) outcomes – hard and soft outcomes.

### Data analysis

Interview transcripts were imported into Nvivo 14 and coded using an inductive-deductive approach using reflexive thematic analysis ( Braun & Clarke, 2021), following the standard six stages: familiarisation with the data; generating and refining initial codes; searching for themes; iteratively refining and defining themes; and writing up.

Listening to the audio/MS Teams recordings enabled familiarisation with the dataset. We kept reflective notes (e.g. thoughts, feelings, questions per interview and across the dataset) and tabulated the main points per interview including context (e.g. work setting), learning (e.g. topic specific) and outcomes (e.g. networking opportunities).

This led to generating initial codes in Nvivo whereby two researchers (Authors 1 and 2) independently coded three transcripts, using a hybrid process of inductive (based on the data) and deductive coding (literature and topic guide) ( Fereday & Muir-Cochrane, 2006). Coding decisions were compared before all relevant data from the remaining transcripts were coded. Each code and sub-code were given a main label and a descriptor in Nvivo. Refining, describing and organising codes was an iterative process, pausing with each iteration to interrogate coding decisions and uncertainties. Each iteration was exported as an MS Word document, maintaining an audit trail. Comments on coding decisions were recorded using the annotations feature of Nvivo; memos were used to record reflections linked to a particular interview and/or (sub-) code. This led to developing themes and theoretical understanding across the dataset. We continued until we reached a level of coding saturation, when no additional issues were identified ( Hennink *et al.*, 2017) and the framework appeared to capture all data.

Thirdly, generating themes, moved the focus from coding each interview to exploring meaning across the dataset. Codes were grouped into potential themes, with some ‘collapsing’ of codes to develop broader patterns of meaning. This involved interpreting the significance of the codes and how they related to each other. For example, we explored within and across different disciplines, roles and settings looking for similarities and differences between researchers, practitioners and care experienced members. We used mind maps to help us interrogate the data and our decisions.

Fourth, reviewing themes involved a recursive review including refining the boundaries of each theme (what was or was not included), in relation to the coded data items and the dataset. This involved combining, splitting and discarding themes to develop a coherent overview of the dataset that accurately reflected the findings and provided the most apt interpretation of the data in relation to the research question.

Fifth, defining and naming themes aimed to capture the essence of each theme and differentiate from other themes. Ongoing analysis refined the focus of each theme, how they interrelated and contributed to the overall narrative. Lastly, writing up the data involved presenting the findings as a coherent whole and in relation to the research question ( Braun & Clarke, 2021).

An additional step was to map the results onto Cooke’s (2005) six principles of research capacity building. We tabulated each sub-theme against each principle, looking for supporting (and refuting) evidence and then developed this into a diagram to illustrate the relationships.

To benefit rigour, Guba and Lincoln’s (1982) concepts of trustworthiness were used. Strategies included maintaining an audit trail; regularly questioning decisions; using reflexivity to challenge assumptions and expectations; comparing across transcripts; analysing instances that did not appear to fit with the majority ( Bazeley, 2013); discussing with other team members; and maintaining an audit trail ( Bazeley, 2013).

### Ethics

Ethical review and approval for this study was granted by Staff Review Committee, Division for the Study of Law, Society, and Social Justice, The University of Kent. The study was approved on 10/10/2022 reference number 0708. 

## Results

### Interview participants

Twenty-one interviews, including one care experienced dyad (married couple, E03) were carried out. Of those, some had more than one role, for example professional and caring responsibilities. Table 3 provides an overview of who was interviewed, which COP they attended, regularity of attendance and who presented at a COP.

**Table 3.** Summary of participants’ role and COP involvement.

Participant id & role	Attended workforce COP	Attended complex needs COP	Regular attendance	Intermittent attendance (or stopped)	Presented at a COP
**P**rofessional ( **P**): practitioners, managers or strategic role; organisation - local authority, RDN/NIHR or independent sector					
P01	✓		✓		
P02	✓	✓	✓		
P03	✓	(✓)		✓	✓
P04		✓	✓		✓
P05	✓	✓		✓	
P06	✓	(✓)	✓		
P07		✓		✓	✓
P08		✓		✓	✓
P09	✓	✓		✓	
P10	✓	✓		✓	
P11	✓	✓		✓	
**R**esearchers ( **R**): in social care &/or with role in research & development					
R1	✓			✓	✓
R2	✓	✓		✓	✓
R3	✓	✓	✓		✓
R4	✓	✓	✓		✓
R5	✓	✓	✓		
Care **E**xperienced members ( **E**): informal carers and those who draw on care/support					
E01	✓	✓	✓		
E02	✓	✓	✓		
E03	✓	(✓)		✓	
E04	✓	✓	✓		
E05	✓	(✓)	✓		
Total:	18	15 (19)	11	10	8

(✓) – attended occasionally but predominantly went to the other COP

***1.   A shared endeavour – fostering an inclusive, collaborative learning environment***

This theme captures how the COPs fostered an inclusive, collaborative space for learning. Participants described the COPs as genuine communities where diverse voices, including researchers, practitioners, and care-experienced individuals, were equally valued. The broad range of topics and flexible structure enabled engagement across disciplines, roles and settings.

**a)   Broad topics and an inclusive learning environment**

Both COPs were regarded as communities and the ‘community element’ remained at the core of both groups (P03). Most respondents liked the variety of topics and noted that it allowed flexibility to invite a wide range of speakers and to address a wide range of topics within the theme’s remit. We expected a clear differentiation between the COPs but this was not evident in the data. Many participants regarded the COPs as two interlinked areas, with blurred boundaries:

It's very difficult to categorise them either as complex needs or the workforce, they straddle both areas (E02)

More importantly, the COPs brought researchers, practitioners and care experienced members together as equal partners:

It's good we all get a chance to speak (…) so we can share the points of view. So yeah, it's not dominated by one person (…) there's an opportunity for people to participate and that's what I think is inclusive. (E05)

Practitioners and researchers commented on the benefits of an inclusive space:

I think particularly for some of the people with lived experience that attended, it was amazing how involved everyone was and you always gave people so much time and showed so much respect (…) it felt like a very safe and inclusive forum, which I think is a really special thing in its own right (R04)

Care experienced members also commented on feeling valued, as equal partners, without sense of hierarchy:

That group, it does stop everyone not to have a badge saying we are social workers and we are doctors and we are disabled people, we are same in that group that is [a] beautiful thing itself (E04, uses a computer to communicate)

**b)   Thirst for capturing, co-producing and contributing to knowledge**

There were different views about how to capture topic specific learning ranging from suggestions that we should have kept a log to the individuality of learning, depending on context, discipline and personal perspectives:

We move on from one angle to another very easily, and many people might find different elements in that session useful, or find different sessions useful, so I feel it will be very hard to capture it as a log or something like that. (R03)

Care experienced members were explicit in their quest for knowledge, sharing learning and desire to contribute to improving practice:

I always come away finding that I've taken something away of something interesting or something else I could read about (…) I think it is making a difference because people are talking about things and if they're going to different places of work or groups wherever they are discussing. (E05)

This led to examples of how topic specific information was shared beyond the COP with colleagues, family and friends. Examples demonstrated how the COPs were closely linked to practice, for example passing on information to staff in a care home, and ‘discussions about the moral injury’ (E05) with a social work friend/colleague.

***2.   Shared learning - within and beyond the COPs***

This theme explores how learning generated within the COPs was shared and transferred across organisational boundaries, highlighting both the opportunities for collaborative growth and the cultural and structural barriers that inhibited wider dissemination and uptake.

**a)   Promoting shared learning within the constraints of the prevailing culture**

There were several comments around the ‘immense pressure time wise’ (P06) staff were under, such that even essential continuing professional development was hard to manage. This was in the context of an organisational culture not conducive to attending COPs:

Overall, it needs to be more of a culture change within social care organisations that practitioners feel they have permission and are encouraged to do this. It’s hard enough to break down that wall of them knowing about it, but then once they do, it’s that idea of permission and the idea of, “I’ve got too much work to do.” (P03)

Comments on the constraints of organisational culture were countered by the need to learn and innovate and demonstrated the impact of tacit learning. Practitioners valued the opportunities that the COPs provided but lack of hard outcomes made it difficult to challenge the status quo:

There might be mechanisms to take tangible outcomes and actually implement those but I think a lot of the time, it's that kind of softer influence that it has around just a raising awareness maybe you know, because of that, do you then develop a bit more of kind of an innovative mindset? Does it make you a bit more open to hearing about opportunities or getting involved in other opportunities around research or embedding things in your own practice? (…) It's almost about changing the culture, isn't it? (R04)

Even so, the prevailing view was that senior management needed to explicitly endorse and prioritise research (and COP attendance) but did not:

They're always in crisis management, aren't they? But it's a false economy not to find time to do it. So I just think it should come down from the higher managers downwards. So it should be, you know, we need to find a time to do this, to build the evidence, need to have applied and embedded research. (P07)

However, there were practitioners who attended COPs regularly and the local authority’s Practice Development Team for Adult Social Care explicitly stated that they were developing a COP for social care practitioners to promote continuing professional development, modelled on our COPs (P09). 

**b)   Learning through building relationships and a shared ‘language’ of research**

Reflecting on each other’s experiences helped develop relationships across roles and settings, providing opportunities to bridge silo thinking:

Well, as a result of these COPs, I think that certainly relationships between academia, frontline delivery and people, experts by experience, I think that there is a better relationship there, which is vital (…) it gives another perspective (P04)

There were discussions around the importance of challenging each other’s attitudes and how this (tacit) learning was important, albeit hard to evidence:

I would have felt that I had a really good experience in there and that I know it would have an impact on my thinking and my practise going forwards, yeah. I might find that difficult to quantify and explain to my immediate manager or my grandparent manager, or anyone higher up. (P10)

Many participants, researchers included, commented on the need to avoid using jargon within COP meetings (and more widely) because each sector (health, social care and research) used different terminology, with health and social care being ‘two very different worlds’ (P06):

I think sometimes that's about language and about not understanding each other. I don't think we always understand research speak and I think researchers don't always understand social care language, and it's a very difficult sector to communicate with. (P01)

While not explicit, this relates to avoiding language that excludes and suggests epistemic superiority. Other comments reflected on the need to learn the terminology of the other ‘camp’ reflecting an ongoing silo between health and social care:

I can't explain it and it's really hard to put a finger on it but having a foot in each camp right now and it does feel like two separate camps, it shouldn't, but it does. (R05)

Linked with this was ensuring the COPs catered to everyone’s level of research understanding, relating back to inclusivity:

There needs to be enough of a kind of shared understanding and where you've got individuals who've got different levels of prior research, knowledge and experience, it's quite difficult to do that. (R02)

All participants were asked what research skills they had learnt from the COPs but most were unable to identify specifics. Some, including care experienced members, already had research training (e.g. Masters) and/or were learning research skills through another strand of the Partnership (e.g. Fellowship funding for research/training) so were unable to directly attribute learning to COPs. Care experienced members identified learning about research terminology, using research to learn about a topic and how to write a lay summary.

***3.   Shared impact - potential for influencing policy and practice***

This theme explores how COPs foster opportunities for networking, validating research ideas, and embedding learning into practice, with aspirations to influence policy and service delivery, even when such impacts are difficult to evidence in traditional ways.

**a)   Networking and developing knowledge-exchange collaborations**

Networking was frequently cited as an outcome, with practitioners valuing better connections whether any tangible outcomes were identified or not:

It was also good for networking opportunities. So getting to know peoples’ names, contact details (…) when I came back from maternity leave, I did touch base with a few people just to, you know, get my head back in the game (P05)

For presenters, it was an opportunity to raise awareness of their role, or ‘brand awareness’ (P08) as well as networking:

Being part of the COPs introduced me to individuals and organisations that I can then refer to, let's say, a care home who's interested in doing some research and partnering them up (P08)

Similarly, researchers valued networking opportunities across disciplines and settings, within and beyond the COP, enabling them to make new contacts:

I met also, I’d say, a good and collaborative community, which is rare to find, because these collaborations between practitioners, members of the public and academia are not easy to build. (R01)

Researchers also valued feedback from care experienced members to ensure that ideas were relevant and meaningful to them. This was reflected in comments about having research ideas validated, or endorsed, by COP members:

It was a validation and the endorsement. This is important, you have your own research questions and you think this is the right thing to do (…) but you do in isolation, so you don't really have a chance to talk about it with other people too much (…) that's [an] important thing for me, is together validation that we are on the right tracks (P07)

**b)   Embedding learning into practice and influencing policy**

There were concrete examples of how information was shared beyond the COP with partner organisations, with the potential to influence practice at both individual and a service level:

I personally found the community of practice really useful (…) I was afterwards able to, decide (…) whoever was presenting at that particular webinar, whether it was relevant for me to share information on that with the sector because it may be useful for them. (P06)

From care experienced members’ viewpoint, the COPs enabled researchers to focus on what mattered in a more tangible sense, with aspirations of changing practice:

The COPs were about real issues and how important it was to face up to some of the issues in them and analyse them. You had the motivation to see it all the way through and to be trusted in it. (E02)

Although too early to evidence, participants had long-term aspirations that what happened within the COP would reach further into partner organisations and potentially inform policy:

The outcome of the COPs meant that I was able to share information and it is, bringing things to people's forefront of their mind, that in time (…) does start to inform policy. (P06)

Participants acknowledged how hard it can be to evidence changes in practice or policy but that this did not preclude the need to try:

Sometimes you just have to do things because they're good in themselves and it's a drip, drip, drip. You're not going to be able to measure things in a quantitative way because there is no way to do that. (P09)

However, linking to sub-theme 2a, the limited support of senior management, largely due to heavy workloads, several practitioners also commented that they did not feel senior management necessarily supported COP attendance, particularly given the difficulty of evidencing tangible outcomes of social learning:

It might be very difficult for them [management] to understand the value that I might be directly getting from spending an hour, hour and a half in such a place because literally nothing can appear to come out of it for them. You know, I might believe that I have that the quality of my experience or understanding and therefore practice would have improved but how can I show that and how do they know that? (P10)

**Figure 2.**
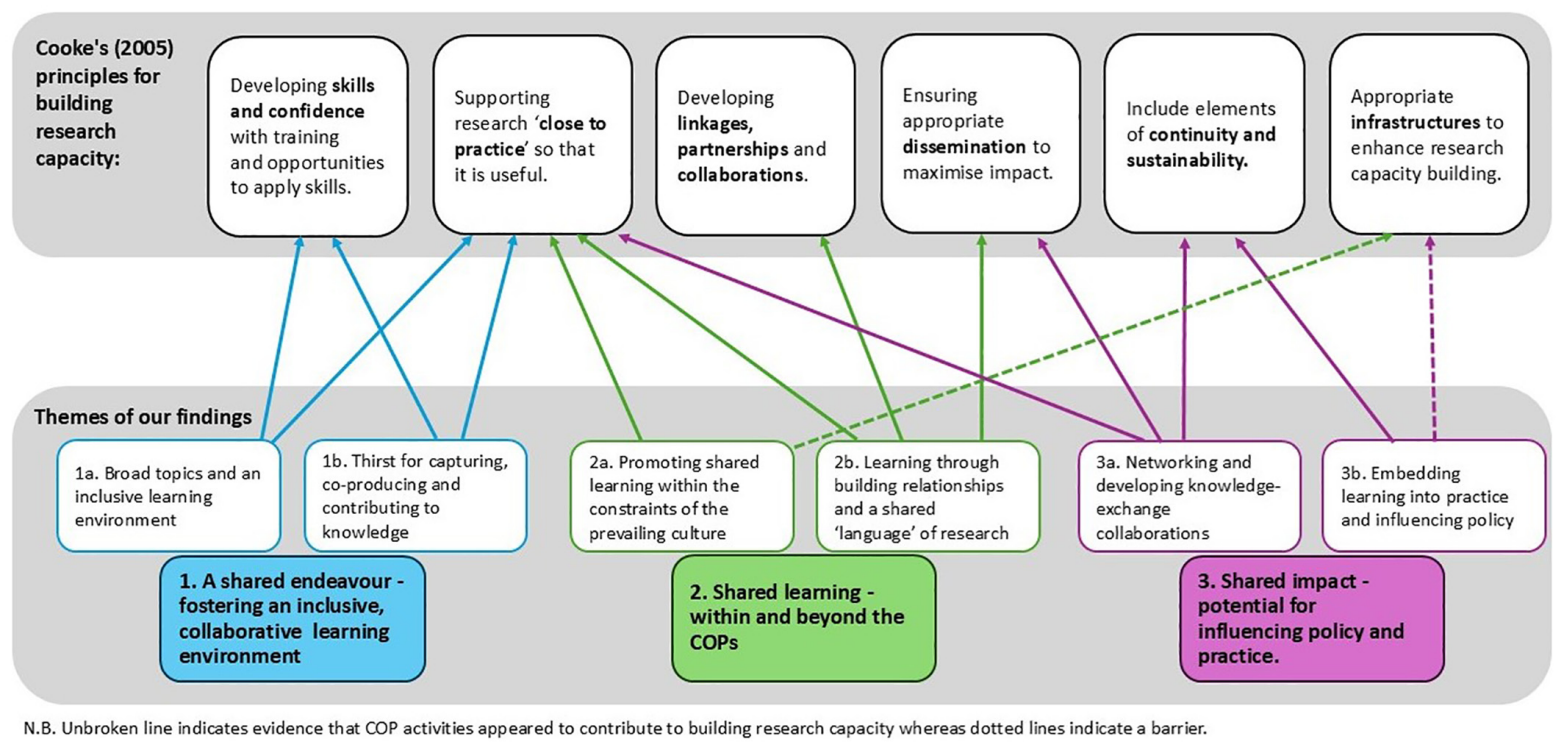
Building research capacity: mapping onto Cooke’s (2005) principles.

Most evidence related to individual and team-level capacity, such as increased confidence, knowledge exchange, and collaborative opportunities. Organisational-level impact was less evident, reflecting broader challenges in embedding learning cultures within adult social care settings. Figure 2 demonstrates the inter-relationships between Cooke’s (2005) principles and the findings.

## Discussion

This paper described the co-development and delivery of two CoPs within the Kent Research Partnership, exploring members’ experiences, perceived benefits, and the COPs’ contribution to research capacity building in adult social care. It also identified key facilitators and barriers to their implementation and sustainability. A central strength of the COPs was their inclusive and egalitarian ethos, which empowered care-experienced members to contribute meaningfully across a broad range of topics. Participants expressed a strong desire to share learning beyond the COPs, demonstrating a commitment to improving practice and achieving the aims they had initially anticipated (see Table 1).

### Learning through participation and social interaction

While the COPs were structured around two thematic domains - ‘Complex Needs’ and ‘Workforce’ - care-experienced members found these boundaries somewhat artificial. This suggests that although the domains reflected practitioner concerns, what sustained the COPs was the social space they created for enriched, less formal learning ( Pyrko *et al.*, 2017). For practitioners and researchers, the distinction between the two COPs was meaningful, reflecting familiar categorisation of need, client groups and professional issues. In contrast, care experienced members did not experience this divide as clearly, arguably because the workforce engages holistically with individuals who have social care needs, making these issues inherently interconnected.

Explicitly evidencing learning proved challenging due to its tacit and contextual nature, yet participants described how they applied and disseminated knowledge informally within their workplaces, families, and wider networks. This resonates with Reed *et al.*’s (2010) argument that to be considered social learning, a process must: demonstrate that a change in understanding has taken place in the individuals involved; demonstrate that this change goes beyond the individual and becomes situated within wider social units or communities of practice; and occur through social interactions and processes between actors within a social network. We found evidence of all three aspects, particularly in participants’ reflections on challenging assumptions, sharing insights, and building networks.

### Sustaining Communities of Practice

Pyrko *et al.* (2017) emphasised that the vitality of COPs lies in their ability to “think together” about real-life problems that people genuinely care about. This collective thinking was evident in our data, where mutual engagement and shared purpose sustained the COPs beyond their original thematic boundaries. Drawing on Wenger’s original discourse ( 1998), Iverson and McPhee ( 2002; 2008) argue that COPs require nurturing to embed knowledge exchange processes. Our findings support this view: although COPs evolved beyond their initial domains - developing malleable boundaries and allowing flexible, though still relevant, topics within adult social care - they continued to function as vibrant, inclusive learning communities.

The COPs created a valuable space for practitioners to develop relationships across settings and foster ‘soft’ learning skills. However, many practitioners perceived that senior management struggled to recognise the value of dedicating time to COP participation, particularly within a culture shaped by significant time and resource constraints and long-standing workforce shortages. This perception echoes Howlett, Arthur and Ferreira’s (2016) evaluation of COPs in education, where poor morale and a sense of institutional indifference led academics to disengage from COPs. Yet, such disengagement often overlooks the broader structural and systemic realities that inhibit learning and participation ( Howlett *et al.*, 2016).

Gray *et al.* (2010) note that COPs naturally align with the ethos of social work, which emphasises autonomy, self-direction, and empowerment. However, they also argue that policy initiatives—both past and present—have done little to foster a culture of learning. Our data suggest that the wider social care context, marked by chronic underfunding and workforce pressures exacerbated by the COVID-19 pandemic, underpins many of the perceived organisational shortcomings. During the lifespan of the COPs, the host organisation underwent restructuring and inspection against new regulatory standards ( Care Quality Commission, 2023), contributing to low morale and a perceived lack of support among staff.

Interestingly, this perception persisted despite senior managers expressing support for the COPs and the local authority continuing to invest in both the COPs and the Research Facilitator role beyond the formal end of the Partnership. This raises questions about the role of perception and professional conscience—many practitioners (and researchers), struggle to prioritise their own continuing professional development (CPD) amidst competing demands.

Gray *et al.* (2010) also suggest that COPs may serve as a valuable learning tool for first-line managers, enabling a values-driven approach to creating a rewarding team environment, even within organisations that are less responsive to practice development. As our findings confirm, given many comments from practitioners and researchers about the value of a shared egalitarian space to learn from care experienced members, social work and social care share a strong value base centred on empowerment. COPs offer a mechanism for practitioners to bring their social awareness into collaborative learning, fostering skills and understanding through groupwork. However, in our study, this potential was constrained by the organisation’s limited capacity to support and embed such learning in a sustained way, reflecting wider constraints and challenges endemic in the social care landscape.

### Building research capacity: networking and collaboration

A key strength of the COPs was their potential for bringing in connections through networking, and developing opportunities for shared learning and collaboration, and consequently increasing the prospect of introducing new ideas into practice. There was evidence supporting all bar one (infrastructure) of Cooke’s (2005) principles to enhance research capacity building. Since the data was collected, the local authority has committed to sustain the Researcher in Residence’s role as a research facilitator within the organisation and established an internal COP for practitioners.

Practitioners described the COPs as hubs for expanding their research engagement and building partnerships. This mirrors findings from El-Amiri *et al.* (2020); Padilla & Kreider (2020), and Gullick & West (2016), who highlight the role of COPs in fostering interdisciplinary collaboration across sectors and enhancing research capacity across sectors. In our study, COPs provided a platform for practitioners to connect with others, share ideas, expand their networks and initiate research-practice partnerships.

### Evidencing impact

Participants noted that while COPs influenced individual and service-level practice, evidencing their impact, particularly at policy level, remained challenging. This reflects broader critiques of COPs’ evidence base, especially regarding how new knowledge is embedded into practice ( James-McAlpine *et al.*, 2023). Evidencing impact of the COPs is not a phenomenon isolated to adult social care but is prevalent in assessing their impacts more broadly for instance in the healthcare sector ( Ranmuthugala *et al.*, 2011). Ranmuthugala *et al.* (2011) argue that measuring COPs’ impact requires baseline indicators and tailored evaluation strategies yet identifying quantitative indicators is challenging. Our findings suggest that while COPs hold promise for capacity building and practice improvement, there remains a gap in robust methods to assess their long-term impact.

### Study limitations

This is a small qualitative evaluation of two COPs in a region of South-East England and thus lessons learned may have limited generalisability. We are not claiming data saturation and may have identified further nuances had we been able to interview more widely – it was particularly hard to recruit frontline staff and researchers. Some participants found it hard to remember the specifics of the COPs they attended, such that their comments were generic and analysis had to take this into account.

## Conclusion and implications for practice

This study demonstrates the value of Communities of Practice (COPs) in building research capacity and fostering inclusive, collaborative learning in adult social care. The COPs were well-regarded as vibrant and collegial forums for sharing learning, developing ideas for practice and research, and strengthening connections across roles and sectors. Care-experienced members played a central role, offering unique perspectives that helped practitioners connect with their social awareness and reflect more deeply on their practice.

To maximise the potential of COPs, it is essential to carry out preparatory work to assess interest, clarify expectations, and co-design the structure, including format, frequency, and aims. Establishing shared understanding of what a COP can deliver is key to ensuring relevance and engagement. However, sustained participation requires organisational support, both in enabling attendance and in promoting the wider dissemination of learning. While practitioners and first-line managers appreciated the informal, experience-based learning that occurred within the COPs, they felt that senior leadership did not actively support their participation. However, given that senior managers from the local authority had invested in the Kent Research Partnership, this perception may reflect broader issues such as low morale or a disconnect between frontline staff and leadership, rather than a lack of support in principle.

A key strength of the COPs was their ability to foster networking and collaboration, support shared learning and thus increase the likelihood of introducing new ideas into practice, potentially influencing policy. Yet, as in other sectors such as healthcare and the voluntary sector, there remains a gap in robust methods for evidencing the impact of COPs. Future initiatives should consider how to embed COPs within service structures and develop meaningful ways to assess their contribution to practice, policy, and organisational learning.

## Data Availability

The qualitative data gathered during this evaluation has not been deposited in any public data repository. Thus, access to the interview transcripts is restricted: the institutional ethical body that reviewed this study did not grant consent to public archiving of the data, and therefore participants in the study were not asked to consent to public archiving of their data. However, should any researchers be interested in accessing the data, they may contact the corresponding author via written request (Dr Vanessa Abrahamson, v.j.abrahamson@kent.ac.uk), clearly outlining the intended use of the data, the expected duration of their research, and the institution that will oversee governance procedures for the project. Requests must be accompanied by documentation of ethical approval relevant to the proposed study. Access may be provided depending on the specifics of the request and following review by our institutional ethical body. Figureshare: Kent Research Partnership - Communities of Practice evaluation, November 2025. Available at: https://doi.org/10.6084/m9.figshare.30774806 ( Abrahamson, 2025). This contains the following extended dataset: Topic guides for COP facilitators Topic guides for practitioners and researchers Topic guides for care experienced members participant information sheets consent form. Data are available under the terms of the Creative Commons Attribution 4.0 International license (CC-BY 4.0).
