# Supplementary material for: Using communities of practice in adult social care to build research capacity and foster best practice: a qualitative evaluation
Source: NIHR Open Res. 2025 Dec 19;5:124. [Version 1] doi: 10.3310/nihropenres.14134.1 (PMC12790595; doi:10.3310/nihropenres.14134.1)
Supplement: Peer review history

# Reviewer report: Reviewer response for version 1

**DOI:** 10.3310/nihropenres.15377.r39064

**Published:** 2026-02-03

**Authors:** Harriet Hunt

**Affiliations:** 1 University of Exeter, Exeter, UK

The authors provide an interesting and detailed summary of their evaluation of the role of Communities of Practice in adult social care in building research capacity and improving best practice, focussing on two example COPs. Whilst there are some areas that could be elucidated or developed further (and which I have commented on below), overall this is a useful and additive account of their evaluation and should be helpful for people in the field.

 The title is suitable and accurate.

 One overall comment on the shortening of Community/ies of Practice to COP/s; within the Background and supporting documents, initially this is shortened to CoP – which is the most familiar shortening of Communities of Practice within the wider literature. After the first use, however, the shortened version is COP. I found this a bit of a distraction, and while not inherently wrong, I’d suggest reverting to the conventional shortening of CoP for consistently, ease of reading, and – if relevant – accuracy in digital indexing.

 The abstract is fair and accurate. In the Conclusions, the only reference to barriers was neutrally worded: “Time constraints and organisational culture affected frontline social worker participation, despite a recognised need for innovation.”. It would be more helpful for the reader if ‘affected’ could be more directive, e.g. ‘restrained’ or ‘limited’.

 In the Introduction, the authors state that “Communities of Practice (COP) have largely been absent as a forum for learning for people with lived experience”. It would be helpful to have references here.

 The Background deals comprehensively with the theoretical basis of this research, strongly grounded in Wenger 1998 and 2002. It is reasonable that detail on the establishment of the COPs is referenced elsewhere and fundamental details are given in the text. One section could be clarified – “Fifty-one COPs, including three joint ones…”. Throughout the manuscript, COPs are referred to as a conceptual entity (i.e. two COPs), rather than referring to each individual meeting as a COP. Here, however, this seems to have been confused, and the authors refer to the number of meetings. This would benefit from greater clarity in wording. The authors also state “Monthly meeting attendance was smaller, between 7–23 attendees per meeting for Complex Needs and 8–28 for Workforce meetings; joint COPs had larger attendance of 30–35.” This is a substantial range but is not revisited in the rest of the manuscript. Given an explicit focus on barriers alongside facilitators under aim 4, this would be worthy of further discussion.

 Within the Methods, the study design was thin on detail and would benefit from more description of the interview methodology. Similarly, it might be helpful to expand detail on participants’ demography to get a fuller sense of people involved in the COPs. NVivo is mistyped as Nvivo. The version of NVivo used is reported as v14 once but might benefit from the software reference alongside. The authors could also usefully add who on the team (Author numbers sufficient) carried out the interviews, transcription, and uploading into NVivo. Description of qualitative analysis is helpfully detailed.

 Table 3 is a helpful addition for readers. It is noteworthy that no E members presented, and this would be interesting to explore further in the text. This also prompted me to question the author statement that “the COPs brought researchers, practitioners and care experienced members together as equal partners” – I do not feel this conclusion is explicitly supported and would instead echo the quote that it felt like an inclusive space for one of the Care Experienced interviewees. The quotes and thematic results feel credible and additive, but there is a noticeable gap in the discussion of barriers to implementation, running and sustainability of COPs. This would be particularly welcome to explore in detail given sparse existing evidence in this area, and the conventional positivity bias which inhibits this form of enquiry.

 The Discussion and Conclusion are well written and logically flow from the Results.

 In closing, I’d like to thank the authors for their diligent and thoughtful work that has produced an insightful evaluation of COPs in adult social care, with learning relevant beyond this field.

Is the work clearly and accurately presented and does it cite the current literature?

Yes

If applicable, is the statistical analysis and its interpretation appropriate?

Not applicable

Are all the source data underlying the results available to ensure full reproducibility?

Yes

Is the study design appropriate and is the work technically sound?

Yes

Are the conclusions drawn adequately supported by the results?

Yes

Are sufficient details of methods and analysis provided to allow replication by others?

Yes

Reviewer Expertise:

My expertise is in social and green social prescribing and the evaluation of community- and nature-based interventions within health and care systems. I use mixed methods, including evidence synthesis, qualitative research, and service evaluation, working across primary, social, emergency, and public health settings with diverse and underserved populations.

I confirm that I have read this submission and believe that I have an appropriate level of expertise to confirm that it is of an acceptable scientific standard.

# Reviewer report: Reviewer response for version 1

**DOI:** 10.3310/nihropenres.15377.r38947

**Published:** 2026-01-16

**Authors:** Kathryn Almack

**Affiliations:** 1 University of Hertfordshire, Hertfordshire, UK

The introduction starts by saying that ‘until recently’ Communities of Practice have largely been absent as a forum for learning for people with lived experience – some indication of what ‘recently’ means would be helpful. Then, in noting recent developments that have seen a proliferation of COPs that have enabled and encouraged idea exchanges and shared learning between these groups – some examples or references would also be helpful.

 The concept of COPs and Wenger’s seminal work is important but could be expanded to deepen the understanding of COPs, which may vary in purpose, structure, facilitation and organisation – see for example Elbrink et al (2024). How Communities of Practice Generate Knowledge Translation Outcomes to Support Public Health Issues: A Realist Synthesis. Health & Social Care in the Community, 2024(1). There is also a taken for granted assumption that COPs are a good thing and will lead to positive outcomes.

 The methods section is clear, in particular the analysis section. Potentially some further detail on how the pragmatic research methodology was woven throughout would be helpful – it is mentioned as the methodological approach but could be illustrated how this was applied throughout the study. How many interviews were conducted online / face-to-face – did this impact the quality of data?

 In the findings, were there any data relating to topics and opportunities to speak that picked up on the fact that none of the care experienced members did any presentations at the COPs meetings? This seems an interesting omission, particularly given the quotes on inclusivity and absence of a hierarchy.

 Not all themes provide evidence from the perspectives of all three groups involved (P, R, E members) – I wondered about the reasons behind this as to whether e.g. practitioners had nothing to say on an inclusive learning environment (theme 1) and then in theme 2 (shared learning) the perspectives of care experienced members are largely absent. This leads to an imbalance in evidencing perspectives across all three groups. Could this be explained or addressed by incorporating other quotes?

 These points on the findings seem important to address given the conclusion that a central strength of the COPs was their inclusive and egalitarian ethos.

 It is interesting to note that care-experienced members found the boundaries of the two COPs somewhat artificial – earlier in the paper we read that these members (or other care-experienced individuals were involved in identifying the focus of the COPs but to what extent were their voices influential in deciding the thematic domains?

 I welcome the discussion on sustaining COPs but this section does veer off into a discussion of value rather than sustainability. This section could bring in maintaining momentum as an element of sustainability. I noted earlier that attendance of the COP meetings varied between 7 to 28 and it was interesting that the joint COPs had higher attendance. Is there a way to provide further detail of attendance – did this drop off to 7 or 8 or was attendance variable across the meetings? How might COPs be sustained when set up – to some extent – artificially i.e. as part of a project rather than organically emerging. Is there, in any case, an argument for COPs to be time-limited?

 Overall I enjoyed reading this paper and it offers a valuable contribution to the discussion of COPs (and Cooke’s framework) applied to social care settings.

 Minor points:

 Just for clarity state that there were  51 meetings across the two COPs (otherwise it can read that there were 51 COPs)

 Sometimes COPs is COPS

Is the work clearly and accurately presented and does it cite the current literature?

Yes

If applicable, is the statistical analysis and its interpretation appropriate?

Not applicable

Are all the source data underlying the results available to ensure full reproducibility?

No

Is the study design appropriate and is the work technically sound?

Yes

Are the conclusions drawn adequately supported by the results?

Yes

Are sufficient details of methods and analysis provided to allow replication by others?

Yes

Reviewer Expertise:

Research capacity building in social care

I confirm that I have read this submission and believe that I have an appropriate level of expertise to confirm that it is of an acceptable scientific standard, however I have significant reservations, as outlined above.

# Reviewer report: Reviewer response for version 1

**DOI:** 10.3310/nihropenres.15377.r39060

**Published:** 2026-01-10

**Authors:** Chinwe U. Nnama-Okechukwu

**Affiliations:** 1 University of Nigeria, Nsukka, Nigeria

Chinwe U. Nnama-Okechukwu

 Department of Social Work, Faculty of the Social Sciences,

 University of Nigeria, Nsukka

**Opening comment**:

 This is an interesting study titled *“Using communities of practice in adult social care to build research capacity and foster best practice: a qualitative evaluation.”* I have carefully reviewed the manuscript and would like to offer the following feedback.

**Manuscript title**:

 The title is appropriate

**Abstract:**

 The abstract is succinct and clearly informs the reader about what to expect in the paper. It would be beneficial to include a brief implication for social work practice in the concluding part of the abstract.

**Plain language summary:**

 Well written with clear findings

**Introduction:**

 The introduction is well structured and clearly identifies a gap in knowledge. It is interesting to note that authors used the opening section (Introduction) to explains Wenger’s original discourse (1998) on how communities of practice [COPs] foster coherence within practice communities through mutual engagement, negotiation of joint enterprise and a shared repertoire, I will suggest that the authors strengthen the argument by incorporating more recent literature that builds on Wenger’s foundational work. This is very important to avoid the danger of a single story.

**Methods:**

 While the pragmatic research method of qualitative research clearly justice the reason for the authors’ research design, I will suggest that the authors provide a very brief example of how pragmatism informed the evaluation process of this study.

 The Patient and Public Involvement (PPI) section provides concrete examples of engagement across research design, data collection, analysis, and knowledge exchange, thereby strengthening the credibility of the study, as reflected in both the methods and results sections. From the findings, it is evident that PPI demonstrates sustained and meaningful involvement. However, PPI should be explained more concisely for readers, who may not be familiar with the concept, particularly social workers outside the United Kingdom who may wish to contextualise it within their own settings.

*Study design*:

 Please clarify what is meant by the phrase *“using in-depth interviews to explore the influence and impact of the CoPs.”* If online interviews were the preferred format for most participants, it would be helpful to indicate how many interviews were conducted online and how many were conducted face-to-face. This clarification was not clearly evident in the manuscript.

*Data collection*: This section is clear and well presented.

*Data analysis*: The data analysis process is detailed and well explained. The research team should be commended for the clear, step-by-step description provided in this section. However, further clarification is needed regarding peer debriefing. Please indicate whether peer debriefing was applied as a method of ensuring rigour in this qualitative study. If so, it should be explicitly discussed in the analysis section. If not, the authors should explain the reason for its omission or identify alternative strategies used to ensure research rigour.

**Result:**

 Did the research team conduct interviews with all 21 participants? Some participants appear to be absent from the results section. For example, participants P02, P05, P08, P011, E01, E03, and R05 were not represented in the narrative, whereas participants P03, P06, P07, P09, E02, E05, and R04 were more prominently featured. Please clarify the reason for this imbalance.

**b)** *Thirst for capturing, co-producing and contributing to knowledge:* The authors should provide a brief explanation of what readers are expected to understand from this theme. I also discovered that the voices of practitioners are largely absent from this narrative. Including at least one practitioner’s account would strengthen this theme, particularly in relation to co-production and knowledge contribution.

**a)** *Promoting shared learning within the constraints of the prevailing culture. *The findings under this section are clearly presented

**a)** *Networking and developing knowledge-exchange collaborations:* If researchers valued feedback from care-experienced members to ensure that ideas were relevant and meaningful, it would be important to include the views of these participants under this sub-theme. Their perspectives were not sufficiently represented in the narrative, yet they are key stakeholders in knowledge-exchange collaborations. I suggest this should be included.

**Discussion**:

 This section is well written. I suggest that the authors include a brief discussion highlighting the key outcomes of the findings as an important area of research focus arising from this study.

 If the findings suggest that the social care context is characterised by chronic underfunding and workforce pressures, exacerbated by the COVID-19 pandemic, the authors should discuss how these challenges can be mitigated to ensure the sustainability of Communities of Practice.

 I agree with the authors write up on the limitation for the study

**Conclusion/ recommendation:**

 This well written

**Reference**:

 Please ensure that references adhere strictly to the journal’s guidelines.

**Concluding Remark:**

 The article is suitable for indexing and should be accepted subject to the authors addressing the suggested corrections.

Is the work clearly and accurately presented and does it cite the current literature?

Yes

If applicable, is the statistical analysis and its interpretation appropriate?

Yes

Are all the source data underlying the results available to ensure full reproducibility?

No

Is the study design appropriate and is the work technically sound?

Yes

Are the conclusions drawn adequately supported by the results?

Yes

Are sufficient details of methods and analysis provided to allow replication by others?

Yes

Reviewer Expertise:

Child and family welfare services

I confirm that I have read this submission and believe that I have an appropriate level of expertise to confirm that it is of an acceptable scientific standard.

# Reviewer report: Reviewer response for version 1

**DOI:** 10.3310/nihropenres.15377.r38940

**Published:** 2026-01-08

**Authors:** David Schmidt

**Affiliations:** 1 The University of Sydney, Sydney, New South Wales, Australia

Thank you for the opportunity to read and comment on this paper which describes the development of two communties of practice in social care and their impact on research capacity.

 The paper overall is well written and enjoyable. I have commented on each section for ease of response if required.

**Abstract and plain language summary **are both well-constructed and informative.

**Introduction **section focuses heavily on individuals receiving social care and the use of COP, but does not mention capacity building. While this information is found elsewhere in the paper, it would be good to refer to it here as this is the introduction to the paper overall.

 The **background **section is well descibed with a logical flow of ideas. Figure 1 provides a visual of the three components of a COP, but it isn't clear what the overlapping portions of the Venn diagram are - is this important?

 One clarification. The first paragraph on Page 5  refers to 51 COPs – is this 51 meetings of the two COPs? I would assume so, but may need to be made more clear.

 The **aims **of the study are clearly presented.

**Methods:**

 Pragmatism as a methodology is a good fit for the topic.

 Methods are well described and steps for ensuring rigour are well established. 

 Results section is comprehensive. The themes presented are distinct, with the slight exception of the final quote in theme 3 which was linked to theme 2a. The quote doesn't seem to support the theme, and would it be better placed under 2a?

 Figure 2 is very helpful, demonstrating the alignment to Cooke's work. The figure slearly highlights the lack of commitment to infrastructure and resourcing.

 The **discussion **section again has a logical flow. The intersect of research priorities and communities of practice is an interesting space, It would be interesting to explore the distiction between a COP with a research focus, to a Community of Inquiry. The works of Martha Cleveland-Innes would be helpful here. The issue of measuring the outcomes of a COP may be addressed using Cleveland-Innes' COI Framework.

**Conclusions **are grounded in the study findings and recommendations are well described.

 I hope these comments are helpful and wish you well with the study.

Is the work clearly and accurately presented and does it cite the current literature?

Yes

If applicable, is the statistical analysis and its interpretation appropriate?

Not applicable

Are all the source data underlying the results available to ensure full reproducibility?

No

Is the study design appropriate and is the work technically sound?

Yes

Are the conclusions drawn adequately supported by the results?

Yes

Are sufficient details of methods and analysis provided to allow replication by others?

Yes

Reviewer Expertise:

Research capacity building

I confirm that I have read this submission and believe that I have an appropriate level of expertise to confirm that it is of an acceptable scientific standard.

## References

[1] : Developing a community of inquiry instrument: Testing a measure of the Community of Inquiry framework using a multi-institutional sample. *The Internet and Higher Education* .2008;11(3-4) : 10.1016/j.iheduc.2008.06.003 133-136 10.1016/j.iheduc.2008.06.003
